# A Comparative Evaluation of Matrix-Assisted Laser Desorption Ionization-Time of Flight Mass Spectrometry (MALDI-TOF MS) and Conventional Methods for the Diagnosis of Dermatophytes

**DOI:** 10.7759/cureus.78344

**Published:** 2025-02-01

**Authors:** Riddhi Singh, Prashant Gupta, Kanupriya Tiwari, Swastika Suvirya, Parul Verma, Gopa Banerjee

**Affiliations:** 1 Microbiology, Ram Manohar Lohia Institute of Medical Sciences, Lucknow, IND; 2 Microbiology, King George's Medical University, Lucknow, IND; 3 Dermatology, King George's Medical University, Lucknow, IND

**Keywords:** conventional methods., dermatophyte, diagnosis, malditof, trichophyton

## Abstract

Background and objective

Dermatophytosis refers to superficial skin fungal infection in humans. The laboratory diagnosis of the pathogens causing dermatophytosis is challenging. In recent years, matrix-assisted laser desorption ionization-time of flight mass spectrometry (MALDI-TOF MS) has emerged as an effective tool for diagnosing and identifying microorganisms. This study aimed to compare MALDI-TOF MS with conventional methods of identification to assess the existing knowledge on the diagnostic capabilities of MALDI-TOF MS for dermatophyte infections. The findings of this study have significant implications for clinical practice, public health, and future research endeavors in dermatophytosis diagnostics.

Methods

This prospective study was conducted in the Post Graduate Department of Microbiology at a tertiary-level teaching Medical University over 12 months, from January 1, 2020, to December 31, 2020. A total of 138 clinical cases among the patients attending the skin OPD with clinical features suggesting fungal skin infections were included. Conventional methods such as microscopy, slide culture, biochemical tests, and culture on Sabouraud dextrose agar (SDA) were used as the reference standard for species identification. ATCC (American Type Culture Collection) strains of dermatophytes were used for quality control. The MALDI-TOF Vitek MS (BioMerieux, Craponne, France) system was employed for comparison, and the agreement between the two methods was determined.

Results

Of the 138 samples, 89 were from males and 49 from females. The majority were taken from the skin (103, 75%) samples followed by nails (30, 21%) and hair (5, 4%). Tinea corporis was the most common clinical type, seen in 81 cases (58.70%), followed by onychomycosis seen in 30 (21.74%) cases, and tinea cruris seen in 16 (11.59%) cases. The most prevalent isolate discovered in tinea corporis was *Trichophyton tonsurans (T. tonsurans)*, followed by *Trichophyton mentagrophytes (T. mentagrophytes)*. Traditional methods and MALDI-TOF MS (Vitek MS) had 100% agreement for *T. tonsurans, Trichophyton rubrum (T. rubrum), and Microsporum gypseum (M. gypseum).* However, there was disagreement *between T. mentagrophytes and Trichophyton interdigitale (T. interdigitale)*, which had 0.00% agreement.

Conclusions

Our research showed that no single diagnostic approach would be optimal for every situation that was examined. However, traditional procedures remain the most dependable and efficient methods for mycological diagnostics. However, MALDI-TOF identification results are available much earlier than those of traditional methods.

## Introduction

Superficial fungal infections in humans are known as dermatophyte infections or dermatophytosis. Dermatophytes enter and multiply in keratinized tissues, including skin, hair, and nails. Direct contact with people, animals, and soil and exposure to contaminated objects, known as fomites, can cause the spread of dermatophytes. These infections are easily diagnosed through medical history, physical examination, and microscopy using potassium hydroxide (KOH). Diagnosis occasionally requires Wood's lamp examination, fungal culture, or histologic examination [1].

Since dermatophytes grow slowly, reliable identification may require several weeks. Moreover, conventional identification techniques cannot be employed with some isolates, which might not produce characteristic morphological features in culture. Cultures are slow-growing, non-proliferative, and often emit pigment. Culture is followed by Lactophenol Cotton Bluemount microscopy, slide culture, and growth on Trichophyton agar [2]. *Microsporum, Trichophyton,* and *Epidermophyton* species are the most common pathogens in skin infections. Occasionally, non-dermatophyte fungi, such as *Malassezia furfur* in tinea versicolor and Candida species, can be responsible for superficial skin infections.

While 16S rRNA and 18S rRNA gene sequencing have traditionally been the preferred methods for microorganism identification, matrix-assisted laser desorption ionization-time of flight mass spectrometry (MALDI-TOF MS) has recently gained recognition as a promising technique for microbial identification and diagnosis. This assay generates spectra comparable to protein fingerprint signatures of microorganisms, which can then be identified within minutes by comparing their spectra with those in a reference spectra database [3]. MALDI-TOF MS has significantly enhanced routine bacterial identification in the clinical microbiology laboratory in the past few years.

The MALDI-TOF MS identification method also promises faster dermatophyte identification [4]. MALDI-TOF can be impacted by critical factors in ordinary laboratory operations, including the sorting of culture media, the duration of incubation, the protein extraction method, the mass spectrometry equipment, and the reference spectra library. Reliable species identification via MALDI-TOF MS should ideally function irrespective of the growth characteristics of fungi. However, the fungal physiology and protein expression profile may be significantly impacted by the culture conditions [5]. Nevertheless, according to some investigators, MALDI-TOF MS bacterial identification is unaffected by culture conditions [6,7].

Since different dermatophyte species may require different treatment plans, it is crucial to isolate and identify the etiological factors causing dermatophytosis. For instance, onychomycosis brought on by non-dermatophyte mold might not react to the usual treatment for dermatophytosis; *Trichophyton tonsurans (T. tonsurans)* in tinea capitis typically requires a shorter course of therapy than that induced by *Microsporum canis (M. canis).* The gold standard for diagnosing dermatophytosis is the isolation and identification of dermatophytes from clinical samples. However, the dermatophyte typically takes a long time to sporulate and grow in culture, thereby causing a delay in diagnosis. Effective care is aided by prompt diagnosis and precise dermatophyte identification. Microbiology laboratories are now frequently using nucleic acid-based molecular techniques to identify fungi quickly and precisely as well as to identify etiological agents straight from the clinical material [8].

The primary objective of the present study involves the observational analysis of dermatophytes via MALDI-TOF MS [Vitek MS; (BioMerieux, Craponne, France)] and the comparative evaluation of MALDI-TOF MS (Vitek MS) and conventional microscopy for the identification of dermatophytes.

## Materials and methods

This prospective observational study was carried out in the Postgraduate Department of Microbiology at a tertiary-level Medical Teaching University over 12 months, from January 1, 2020, to December 31, 2020. This study was conducted in accordance with the ethical guidelines. Informed consent was obtained from all participants, and confidentiality of patient data was maintained throughout the study.

A total of 138 clinically diagnosed skin, hair, and nail infection cases of all age groups and both sexes attending the Skin OPD of our hospital were selected for the study. All clinically suspected superficial fungal infection patients were enrolled. Patients diagnosed with skin diseases other than dermatophytosis and those without consent were excluded. The clinical isolates and ATCC (American Type Culture Collection) strains of *T. tonsurans *(28942), *Trichophyton rubrum (T. rubrum)* (28188),* Trichophyton mentagrophytes (T. mentagrophytes)* (MYA4439), and *Trichophyton interdigitale (T. interdigitale) (*9533) were tested using both conventional methods and MALDI-TOF MS.

Conventional method

After direct microscopic examination, the culture was performed in three media: Sabouraud dextrose agar (SDA) with chloramphenicol 50 mg/L and cycloheximide 500 mg/L, and potato dextrose agar (PDA) and dermatophyte test medium (DTM). These were incubated at room temperature for four weeks and 10 days. A pure isolate was generated by sub-culturing on SDA and PDA media for visual and microscopic examinations of cultures (color and growth pattern) and morphological characteristics for further differentiation. Trichophyton agar, urease test, hair perforation test, and slide culture methods were used to identify dermatophyte species.

MALDI-TOF method

**Figure 1:**
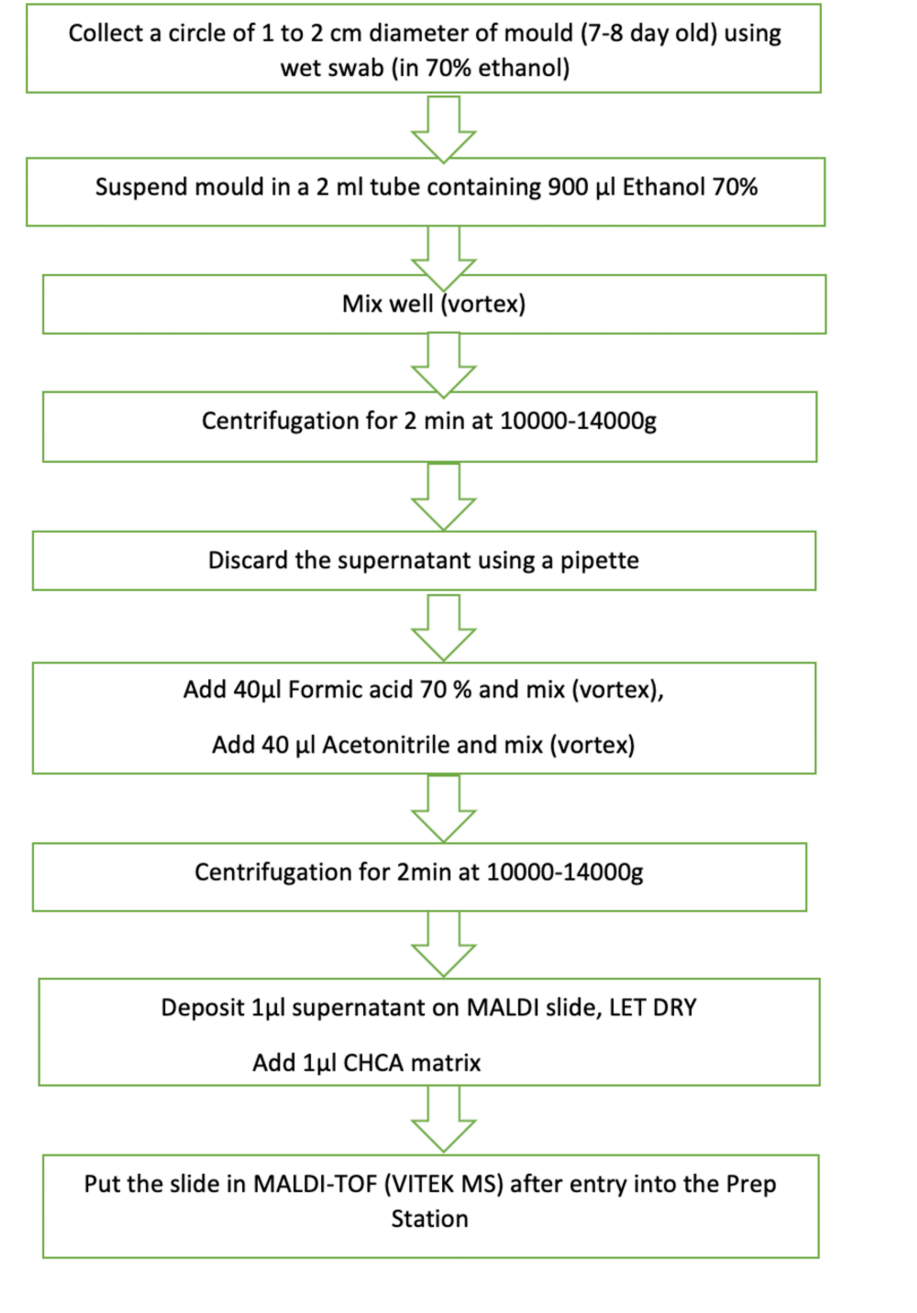
Stepwise flow chart depicting the work flow on MALDI-TOF MALDI-TOF: matrix-assisted laser desorption ionization-time of flight

The MALDI-TOF method for inactivating and extracting mold was performed as follows (Figure 1): The mold was collected using a wet swab moistened with 70% ethanol and suspended in an Eppendorf tube containing 900 µL of 70% ethanol. After centrifugation for two minutes, the supernatant was discarded, and 70% formic acid was added, followed by 60% acetonitrile. The tube was centrifuged again for two minutes. For slide preparation, 1 µL of the extracted supernatant was loaded onto a slide and allowed to dry. Subsequently, 1 µL of CHCA matrix was added. The bacteria/fungi option was selected, and the slide barcode was scanned. After all the required information was entered, the slide was loaded into the machine, and the process was initiated. The completion of the acquisition process was awaited for result interpretation.

Statistical analysis 

The data obtained from the study were analyzed using appropriate statistical methods. The agreement between the two methods was assessed using measures such as Cohen's kappa coefficient. Statistical significance was determined using the chi-square test.

## Results

Of the 138 cases, 35.51% were female and 64.49% male. The cohort's mean age was 32.40 ± 18.02 years, and the majority (26.81%) of patients were aged 21-30 years. Tinea corporis was the most frequently suspected clinical diagnosis (58.70%), followed by tinea cruris. Conventional methods identified a total of 83 dermatophytes as* T. tonsurans *(60.14%), followed by *T. mentagrophytes *(18.84%) and *T. rubrum* (18.11%). The least common was *M. gypseum *(2.90%). Similarly, the most common species identified by MALDI-TOF MS (Vitek MS) was *T. tonsurans *(60.14%), followed by* T. rubrum* (18.11%) and *T. interdigitale* (17.39%).

The comparative analysis of dermatophyte identification using conventional methods and MALDI-TOF MS (Table 1) revealed both concordance and discrepancies. Both methods identified *T. tonsurans* (60.14%),* T. rubrum *(18.11%), and *M. gypseum* (2.90%) with identical detection rates. However, a significant difference was observed in differentiating *T. mentagrophytes *and *T. interdigitale:* the conventional method identified 18.84% as *T. mentagrophytes, *whereas MALDI-TOF MS classified all these as *T. interdigitale *(17.39%). Additionally, MALDI-TOF MS uniquely identified 1.45% of cases as "unidentified." The percentage agreement between the two methods (Table 2) was 100% for *T. tonsurans, T. rubrum, *and *M. gypseum,* but no agreement was observed for *T. mentagrophytes or T. interdigitale.* Cohen’s kappa coefficient (Table 3) revealed strong agreement for M. gypseum (κ = 0.826, p<0.001) but showed variability for other species.

**Table 1:** Dermatophyte identification by conventional vs. MALDI-TOF methods *Statistically significant MALDI-TOF MS: matrix-assisted laser desorption ionization-time of flight mass spectrometry

Conventional method			MALDI-TOF MS			P-value
Species	N (138)	%	Species	N (138)	%	X=54.08, p<0.0001*
T. tonsurans	83	60.14%	T. tonsurans	83	60.14%	
T. rubrum	25	18.11%	T. rubrum	25	18.11%	
T. mentagrophytes	26	18.84%	T. mentagrophytes	0	00.00%	
T. interdigitale	0	00.00%	T. interdigitale	24	17.39%	
M. gypseum	4	2.90%	M. gypseum	4	2.90%	
Unidentified	0	00.00%	Unidentified	2	1.45%	
Total	138	100%	Total	138	100%	

**Table 2:** Percentage agreement between MALDI-TOF MS (Vitek MS) and conventional methods

Species	% Agreement
T. tonsurans	100.00%
T. rubrum	100.00%
T. mentagrophytes	00.00%
T. interdigitale	00.00%
M. gypseum	100.00%

**Table 3:** The assessment of agreement between conventional and MALD-TOF identification methods by using Cohen's kappa coefficient Cohen's kappa coefficient of 0.826 indicates a substantial agreement between the conventional and MALDI-TOF methods in their classification of fungal species. This suggests a high level of consistency and reliability in the identification process. The results demonstrate the effectiveness of both methods and support their use in clinical practice for diagnosing and categorizing fungal infections MALDI-TOF: matrix-assisted laser desorption ionization-time of flight

Comparison			MALD_ID					Agreement	
			M. gypseum	T. interdigitale	T. rubrum	T. tonsurans	Unidentified	Kappa value	P-value
Conventional_ID	M. gypseum	N	4	0	0	0	0	0.826	<0.001
		%	2.9%	0.0%	0.0%	0.0%	0.0%		
	T. interdigitale	N	0	0	0	0	0		
		%	0.0%	0.0%	0.0%	0.0%	0.0%		
	T. mentagrophytes	N	0	24	0	0	2		
		%	0.0%	17.3%	0.0%	0.0%	1.4%		
	T. rubrum	N	0	0	25	0	0		
		%	0.0%	0.0%	18.1%	0.0%	0		
	T. tonsurans	N	0	0	0	83	0		
		%	0.0%	0.0%	0.0%	60%	0.0%		
	Unidentified	N	0	0	0	0	0		
		%	0.0%	0.0%	0.0%	0.0%	0.0%		

Testing of ATCC strains using MALDI-TOF MS (Table 4) demonstrated accurate identification for *T. tonsurans* (ATCC 28942) and *T. rubrum* (ATCC 28188) but misclassified *T. mentagrophytes* (ATCC MYA4439) as *T. interdigitale*. However, *T. interdigitale* (ATCC 9533) was correctly identified. These results underscore the high concordance of MALDI-TOF MS with conventional methods for certain species while highlighting its limitations in distinguishing closely related species like *T. mentagrophytes* and *T. interdigitale*. Enhancements to the MALDI-TOF database are necessary to improve species-level differentiation and overall diagnostic accuracy.

**Table 4:** Results of ATCC strains with MALDI-TOF MS (Vitek MS) ATCC: American Type Culture Collection; MALDI-TOF: matrix-assisted laser desorption ionization-time of flight

Organisms	ATCC	Identified by MALDI -TOF MS (Vitek MS)
T. tonsurans	28942	T. tonsurans
T. rubrum	28188	T. rubrum
T. mentagrophytes	MYA4439	T. interdigitale
T. interdigitale	9533	T. interdigitale

All ATCC strains of *T. mentagrophytes* were identified as* T. interdigitale* by MALDI-TOF MS (Vitek MS) repeatedly five times. 

## Discussion

The prevalence of dermatophytosis in humans has increased dramatically over the last two decades due to poor personal hygiene caused by rising financial expenses and overcrowding. Worldwide travel, migration, and interaction with animals (especially pets) have been added risk factors [9]. Dermatophytes have occasionally been associated with epidemics and self-limiting outbreaks [10]. In our study, tinea corporis was the most commonly diagnosed condition (58.70%), followed by tinea cruris. Similar findings were made by Tahiliani et al. [11], Mathur et al. [12], Kumar et al. [13], and Vyas et al. [14]. This could be attributed to the higher incidence of dermatophytosis in the groin, waist, underarms, and toes due to the high sweating rate in these areas. 

India is predominantly a tropical country with a hot and humid climate. Excessive sweating in areas like the groin, waist, underarms, and toes can be attributed to the higher density of sweat glands in these regions, which are also prone to increased friction and heat retention. The male predominance in this issue may be linked to factors such as the nature of male-dominated physical jobs, which often involve strenuous activities in hot or humid conditions, leading to higher sweat production. Additionally, personal hygiene habits, such as less frequent use of antiperspirants or less attention to managing sweat, may also contribute. Combined with environmental factors like exposure to heat, these elements make males more likely to experience increased sweating in these areas compared to females. The lower incidence in females could be linked to non-reporting due to the prevailing social stigma in rural populations and the poor health-seeking behavior of females [15-17].

Tremendous strides have been made in the identification and diagnosis of dermatophytes over the years. Traditionally, dermatophytes were identified using microbiological and biochemical characteristics [18]. Conventional procedures are time-consuming and require specialized protocols, skills, and experience [19]. On the other hand, DNA-sequence-based identification, the gold standard, is prohibitively expensive and time-consuming in a regular microbiology laboratory [20]. Another potential technology for microbe identification is the fingerprinting of protein extracts using MALDI-TOF MS [18]. Over the last decade, MALDI-TOF MS has gained popularity in clinical diagnostic laboratories for the rapid, simple, and reliable identification of pathogen microorganisms such as bacteria, yeasts, and filamentous fungi [21]. In this context, the present prospective observational study aimed to analyze the efficacy of the identification of dermatophytes by MALDI-TOF up to the species level.

Conventional laboratory tests can take up to one month to correctly identify dermatophytes. A direct microscopic inspection is the first step in the conventional gold standard diagnostic process, followed by three to four weeks of SDA culturing. Additional post-culturing identification utilizing biochemical tests or microculture methods may be required [22]. Since MALDI-TOF MS analysis only needs a small colony, precise identifications can be made in just three to six days, and more specifically, before their distinctive morphological features appear. Hence, the turnaround time for identification is significantly shorter compared to traditional morphological identification [23]. MALDI-TOF MS (Vitek MS) is used in laboratories to classify microbes by comparing their barcodes to those in a library. It offers improved accuracy, lower costs, and faster results. Benchtop platforms are also now available for routine fungus identification. Researchers have also used MALDI-TOF MS to classify human fungal pathogens, and a systematic protocol has been developed for detecting filamentous fungi in routine labs [8,17].

In our study, MALDI-TOF MS (Vitek MS) identified 98.55% (136/138) isolates. Of them, the most commonly found species were *T. tonsurans,* followed by *T. rubrum *and *T. interdigitale.* Our findings are consistent with the results of other researchers [24,25]. However, MALDI-TOF was not able to identify* T. mentagrophytes.* It identified all *T. mentagrophytes* as *T. interdigitale.* ATCC strains of *T. mentagrophytes* were repeatedly (five times) identified as *T. interdigitale* by MALDI-TOF. This issue in Vitek MS may be due to the incomplete reference spectrum library of MALDI-TOF (Vitek MS) [26]. Similarly, Pryce et al. [27] observed two instances of discordant identification results between the phenotypic and its sequence-based identifications: one where *T.* *mentagrophytes* was misidentified as *T. interdigitale* and another involving *Chrysosporium indicum *being misidentified as *T. interdigitale.*

Two dermatophyte species that MALDI-TOF repeatedly failed to identify were later identified as *T. *mentagrophytes by conventional methods. This could be due to inappropriate spot development on the MALDI-TOF (Vitek MS) slide or contaminated/mixed culture growth. The main concern associated with MALDI-TOF MS-based identification of pathogenic fungi is the similarities of the molecular components, which may cause sister species to be indistinguishable from each other. For each MALDI-TOF MS system, the reference database for the coverage of microbial species is the Achilles' heel of the MALDI-TOF MS approach. In light of this, laboratories are recommended to generate and complement the mass spectra for their local main species or strains and record them in the reference commercial libraries [28].

Various subtypes of MALDI-TOF databases are available. Bruker and MALDI-TOF Vitek MS are both widely used techniques for identifying dermatophytes. Both methods offer rapid and accurate identification of dermatophytes based on their protein profiles. However, Bruker's system has a more extensive database, covering a broader range of species and strains, thereby providing better identification accuracy. Bruker Daltonics has been described to provide 100% accuracy, while Vitek MS provided 95.4% for identifying dermatophytes. It also offers excellent reproducibility and user-friendly software. Consequently, Bruker's MALDI-TOF MS system is considered superior to Vitek MS for dermatophyte identification due to its extensive database and superior performance [29].

Limitations

This study has a few limitations. Our study was conducted at a single center. Hence, to gain more accurate data and bypass the possible confounders, we recommend a resilient, multicentre study with a sizeable descriptive sample size and different MALDI-TOF systems by other manufacturers. Apart from this, the MALDI-TOF results can be further confirmed by DNA sequencing.

## Conclusions

Conventional methods for the identification of dermatophytes, while effective, are time-consuming and may be influenced by subjective variations in species identification. On the other hand, the MALDI-TOF MS (Vitek MS) system has emerged as a quick, simple, and economical way for dermatophyte identification, serving as a valuable complement to conventional approaches. However, it is essential to exercise caution when identifying *T. mentagrophytes* by using MALDI-TOF (Vitek MS) due to potential cross-identification with *T. interdigitale*.
